# Differential Regulation of Human 3β-Hydroxysteroid Dehydrogenase Type 2 for Steroid Hormone Biosynthesis by Starvation and Cyclic Amp Stimulation: Studies in the Human Adrenal NCI-H295R Cell Model

**DOI:** 10.1371/journal.pone.0068691

**Published:** 2013-07-09

**Authors:** Sameer Udhane, Petra Kempna, Gaby Hofer, Primus E. Mullis, Christa E. Flück

**Affiliations:** 1 Department of Pediatrics, Division of Pediatric Endocrinology and Diabetology, University Children’s Hospital, Berne, Switzerland; 2 Department of Clinical Research, University of Berne, Berne, Switzerland; 3 Graduate School Berne for Cellular and Biomedical Sciences, University of Berne, Berne, Switzerland; North Carolina State University, United States of America

## Abstract

Human steroid biosynthesis depends on a specifically regulated cascade of enzymes including 3β-hydroxysteroid dehydrogenases (HSD3Bs). Type 2 HSD3B catalyzes the conversion of pregnenolone, 17α-hydroxypregnenolone and dehydroepiandrosterone to progesterone, 17α-hydroxyprogesterone and androstenedione in the human adrenal cortex and the gonads but the exact regulation of this enzyme is unknown. Therefore, specific downregulation of HSD3B2 at adrenarche around age 6–8 years and characteristic upregulation of HSD3B2 in the ovaries of women suffering from the polycystic ovary syndrome remain unexplained prompting us to study the regulation of HSD3B2 in adrenal NCI-H295R cells. Our studies confirm that the HSD3B2 promoter is regulated by transcription factors GATA, Nur77 and SF1/LRH1 in concert and that the NBRE/Nur77 site is crucial for hormonal stimulation with cAMP. In fact, these three transcription factors together were able to transactivate the HSD3B2 promoter in placental JEG3 cells which normally do not express HSD3B2. By contrast, epigenetic mechanisms such as methylation and acetylation seem not involved in controlling HSD3B2 expression. Cyclic AMP was found to exert differential effects on HSD3B2 when comparing short (acute) versus long-term (chronic) stimulation. Short cAMP stimulation inhibited HSD3B2 activity directly possibly due to regulation at co-factor or substrate level or posttranslational modification of the protein. Long cAMP stimulation attenuated HSD3B2 inhibition and increased HSD3B2 expression through transcriptional regulation. Although PKA and MAPK pathways are obvious candidates for possibly transmitting the cAMP signal to HSD3B2, our studies using PKA and MEK1/2 inhibitors revealed no such downstream signaling of cAMP. However, both signaling pathways were clearly regulating HSD3B2 expression.

## Introduction

Steroid hormone production depends on cholesterol and a well described cascade of steroid enzymes that are specifically regulated (e.g. developmentally and tissue specific) to fulfill diverse biological functions [1], [2]. The group of 3β-hydroxysteroid dehydrogenase (HSD3B) enzymes plays a central role early in this cascade for the biosynthesis of mineralocorticoids, glucocorticoids and sex steroids [2]. HSD3Bs are membrane bound enzymes which catalyze two reactions, the conversion of the hydroxyl group to a keto group on carbon 3, and the isomerization of the double bond from the B ring (Δ5 steroid) to the A ring (Δ4 steroid) [1]. These reactions depend on the co-factor nicotinamide adenine dinucleotide (NAD) [3]. The human HSD3B family consists of two members, namely the HSD3B type I (HSD3B1) and the HSD3B type II (HSD3B2). HSD3B1 is mainly expressed in the placenta, the mammary glands, the prostate as well as in peripheral tissues such as the skin and the adipose tissue [4], [5]. The HSD3B1 gene on chromosome 1p13.1 encodes a protein of 372 amino acids [2]. By contrast, HSD3B2 is mainly expressed in the adrenals and the gonads [6], [7], [8]. It is also located on chromosome 1p13.1 and encodes a protein of 371 amino acids which is 93.5% identical with the type I enzyme. In the human adrenal cortex HSD3B2 acts as a Δ5–Δ4-isomerase because it converts Δ5 steroids (pregnenolone, 17α-hydroxypregnenolone, dehydroepiandrosterone (DHEA)) to Δ4 steroids (progesterone, 17α-hydroxyprogesterone, androstenedione (Δ4A)).

The human fetal adrenal does not really express HSD3Bs and predominantly produces DHEA [1]; an exception occurs around 6–12 weeks gestation when timely restricted HSD3B2 expression leads to cortisol synthesis and an inhibition of DHEA production [9]. This specific window of HSD3B2 expression is responsible for safeguarding the female external genitalia from virilization during fetal development [9]. After birth, the definitive adrenal cortex consists of the zona glomerulosa and the zona fasciculata which both abundantly express HSD3B2 for mineralocorticoid and glucocorticoid production throughout life. By contrast, the third and innermost layer of the adrenal cortex, the zona reticularis, slowly develops after birth and becomes functionally active for androgen production around 6–8 years of age during an event called adrenarche [10], [11]. Although the biology of adrenarche is not fully understood, a major characteristic of steroidogenesis at adrenarche is a specific downregulation of HSD3B2 expression in the zona reticularis [12], [13]. Furthermore, specific overexpression of HSD3B2 has been found in the ovaries of women with polycystic ovary syndrome (PCOS) [14], [15], which is the most common endocrine disorder associated with hyperandrogenism and infertility in females [16]. By contrast, deficiency of HSD3B2 through genetic mutations causes congenital adrenal hyperplasia which manifests as primary adrenal insufficiency and disordered sexual development in both sexes causing moderate to severe undervirilization of 46,XY and mild virilization of 46,XX infants [17], [18], [19].

Thus, HSD3B2 and its specific regulation seem to play an important role in biology. However, so far little is known about the exact regulation of HSD3B2. Previous studies have shown that transcription of the HSD3B2 gene is activated by the transcription factor steroidogenic factor 1 (SF1) through stimulation of phorbol esters [20]. It is known that the human HSD3B2 promoter contains four consensus GATA elements which can be activated by GATA-4 and GATA-6 and act in concert with the nuclear receptors SF1 and liver receptor homolog 1 (LRH1) [21]. Also the transcription factor Nur77, also known as nerve growth factor IB (NGFI-B), regulates HSD3B2 transcription in granulosa as well as Leydig and adrenal cells [22], [23]. Nur77 apparently activates the HSD3B2 promoter through a nuclear binding response element (NBRE) site [22], [23], [24]. Previous studies have also demonstrated that prolactin stimulated Stat5 activates the human HSD3B2 promoter [25]. Overall, HSD3B2 seems to be clearly regulated at the transcriptional level while other regulatory mechanisms remain to be elucidated. Therefore, the aim of our study was to further investigate the molecular regulation of human HSD3B2 in the context of adrenal steroidogenesis. For our studies, we used the human adrenocortical carcinoma NCI-H295R cell line as our cell model expressing HSD3B2 [26] and the human placental JEG3 cell line as the cell model expressing HSD3B1, but not HSD3B2 [27]. We studied HSD3B2 transcription, expression and function in NCI-R cells grown under varying culture conditions and under 8Br-cAMP stimulation. Furthermore, signaling studies were performed testing for involvement of cAMP dependent protein kinase (PKA) and mitogen activated protein kinase (MAPK)/extracellular signal-regulated kinases (ERK) in HSD3B2 modulation.

## Materials and Methods

### Materials

To obtain the HSD3B2 promoter luciferase reporter constructs, the human HSD3B2 promoter DNA sequences −101, −170, −301 and −1050 to +53 were PCR amplified from genomic DNA extracted from human adrenal NCI-H295R cells using specific primers (Table 1). Obtained PCR products were cloned into the mammalian expression vector pGL3 basic (Promega, Madison, WI, USA) and the new vectors were confirmed by direct sequencing. Plasmids containing the cDNAs for GATA4/6 and SF1 were available from other projects [27]. Nur77 was cloned from total RNA of NCI-R cells via reverse transcription and specific PCR; the obtained cDNA was then cloned into pcDNA3 (Invitrogen, Life Technologies, Paisley, UK). Mutant constructs were generated by PCR-based site-directed mutagenesis using the manufacturer’s protocol (Stratagene AG, Basel, Switzerland) and the obtained constructs were checked for correct mutations by direct sequencing. Eight-bromoadenosine 3′,5′-cyclic monophosphate (8Br-cAMP), PD98059 mitogen-activated kinase kinase (MEK) inhibitor, U0126 MEK inhibitor and H89 cAMP dependent protein kinase (PKA) inhibitor were purchased from Sigma-Aldrich (Buchs, Switzerland) and dissolved in DMSO. For epigenetic experiments, 5-aza-2′-deoxycytidine (5-aza-dC) and trichostatin A (TSA) were purchased from Sigma-Aldrich and dissolved in DMSO. The chicken peptide antibody against human HSD3B2 was custom made by Genetel Pharmaceutical Co Ltd (Guangdong, China). β-actin antibody was received from Sigma-Aldrich. Secondary goat anti-mouse IgG or goat anti-chicken IgG, both conjugated with horseradish peroxidase were purchased from Santa Cruz Biotechnology (Santa Cruz, CA, USA). Radioactive-labeled [1,2,6,7(N)-3H] DHEA (63 Ci/mmol) was procured from PerkinElmer (Boston MA, USA).

**Table 1:** List of oligonucleotide primers used for cloning, site directed mutagenesis (SDM) and PCR.

**Primers for HSD3B2 promoter constructs (M77144)**			
−1050 HSD3B2 _F		S: 5′- CCG**CTCGAG**AGTGGGAACTCTGTGGGAATAA –3′	
−301 HSD3B2_F		S: 5′- CCG**CTCGAG**ACTTGGAGACTTCTCCCAGTTT –3′	
−170 HSD3B2_F		S: 5′- CCG**CTCGAG**TTCTCTTCCTGTTCCTGG –3′	
−101 HSD3B2_F		S: 5′- CCG**CTCGAG**TTCTGGAGGAGGAGGGAGCAA –3′	
+53 HSD3B2_R		AS: 5′- GTCCC**AAGCTT**AGATTGTTAAAAGCTGGACAGAG –3′	
**Primers for Nur77 cloning (BC016147)**			
Nur77		S: 5′- CCC**AAGCTT**AGCCATGCCCTGTATCCAAGCC –3′	
		AS: 5′- CGC**GGATCC**TCAGAAGGGCAGCGTGTCC –3′	
**Primers for SDM on HSD3B2 promoter constructs** **(M77144)**			
−301 HSD3B2/GATA mutation		S: 5′- CTAAAGCCAAGACTCT**TTAAG**ACACTGTGGCCTT–3′	
−301 HSD3B2/NBRE mutation		S: 5′-GAATTAGAGATATAACCT**AGAATTCA**CTATTATTCTGAGAAAAGG –3′	
−301 HSD3B2/SF1 mutation		5′- GTATGTGGCAGGAGT**TCAATTTAA**TAAGGGCTGAGAC–3′	
**Primers for RT-PCR**			
Nur77 (NM_002135)	S: 5′- ATACACCCGTGACCTCAACC–3′		
	AS: 5′-CTTCTGGAAGCGGCAGAACT–3′		
HSD3B2 (NM_000198)	S: 5′- AAGCTGACTGTACTTGAAGG–3′		
	AS: 5′-GTGTACAAGGTATCACCATT–3′		

Restriction enzyme targeted sequences or consensus sequences are shown in **bold**. Mutated nucleotides are underlined. GenBank accession numbers for source sequences are given in parentheses.

### Cell Cultures, Transfection and Dual Luciferase Assays

Human adrenocortical NCI-H295R cells were purchased from American Type Culture Collection (ATCC; CRL-2128). NCI-R cells were cultured in DMEM/Ham’s F-12 medium containing L-glutamine and 15 mM HEPES medium (GIBCO, Paisley, UK) supplemented with 5% NU-I serum (Becton Dickinson, Franklin Lakes, NJ, USA), 0.1% insulin, transferrin, and selenium (100 U/ml; GIBCO), penicillin (100 U/ml; GIBCO) and streptomycin (100 µg/ml; GIBCO). The serum free starvation medium consisted of DMEM/Ham’s F-12 medium, penicillin (100 U/ml; GIBCO) and streptomycin (100 µg/ml; GIBCO). Human embryonic kidney 293 (HEK293) cells [28] were maintained in DMEM +4.5 g/L D-glucose with 10% fetal calf serum, penicillin (100 U/ml; GIBCO), streptomycin (100 µg/ml; GIBCO) and 1% sodium pyruvate (100mM; GIBCO). NCI-R and HEK293 cells were transfected with the empty vector pGL3 or specific HSD3B2 promoter-reporter constructs and different transcription factors as indicated. Transient transfection was carried out in 12-well plates for 6 hours (Falcon 3043; Becton Dickinson) using Lipofectamine 2000 reagent according to the manufacturer’s instructions (Invitrogen). Per 12-well, the transfection mixture contained 2.5 µg plasmid DNA and 100 ng Renilla luciferase reporter (pRL-TK) (Promega, Dübendorf, Switzerland) for endogenous control. Human placental JEG3 cells were purchased from ATCC (HTB-36™) and cultured in minimal essential medium (MEM) with Earle’s salts (GIBCO), 10% fetal bovine serum, 1% L-glutamine (200mM; GIBCO), penicillin (100 U/ml; GIBCO) and streptomycin (100 µg/ml; GIBCO). Before transfection, JEG3 cells were divided onto 12-well plates at approximately 50% confluency. For transfection, cells were then incubated for 6 h with calcium phosphate precipitates containing the −1050 bp HSD3B2 promoter reporter plasmid DNA and DNA of expression vectors for GATA4/6, SF1 and Nur77 (2.5 µg total DNA/duplicate). Forty-eight hours after transfection, cells were washed with phosphate buffered saline (PBS) and then assayed for luciferase activity using the Dual Luciferase Reporter Assay system and protocol by Promega.

### Western Blot Analysis

For protein analyses, treated cells were washed with ice-cold PBS and harvested in lysis buffer (200 mM Tris-HCl, pH 7.5, 150 mM NaCl, 1 mM EDTA, 1% Triton X-100 and protease and/or phosphatase inhibitors. Lysates were homogenized by a 25G syringe, centrifuged at 13,000×g for 10 min at 4°C and supernatants were collected. Protein concentrations of samples were measured by the DC Protein Assay (Bio-Rad, Hercules, CA, USA). For Western blots, 30 µg protein of total cell lysates was mixed with SDS loading buffer (62.5 mM Tris–HCl, pH 6.8; 2% sodium dodecylsulfate, 10% glycerol, 100 mM dithiotreitol, 0.01% bromophenol blue), heated for 5 min at 95°C, separated on a 10% SDS-PAGE gel and blotted on an Immobilon-P PVDF transfer membrane (Millipore, Bedford, MA, USA) using the semi-dry transfer method. Blocking and staining with antibodies was performed according to manufacturer’s recommendations (Santa Cruz Biotechnology or Sigma-Aldrich). For HSD3B2 Western, membranes were blocked with 10% milk, HSD3B2 antibody was used at 1∶10′000 and the secondary antibody was dissolved 1∶10′000 in Tween 20 tris-buffered saline (TTBS) with 5% milk. Protein bands were visualized by Plus-ECL substrate reagent (PerkinElmer) and exposed on HyperfilmMP (GE Healthcare Limited, Buckinghamshire, UK). When tested with different antibodies, membranes were stripped with 0.2 M NaOH for 30 min and washed with TTBS before restaining. β-actin was used to control for equal loading. For quantification, protein bands were densitometrically measured using Quantity One software (Bio-Rad).

### Steroid Labeling

In whole-cell experiments, steroidogenesis was labeled by adding 60,000 cpm [^3^H]DHEA per 12 well for 60 min. Steroids were extracted from medium as described [29], and products were separated by thin layer chromatography (TLC) (Macherey-Nagel, Düren, Germany) using the chloroform:ethylacetate (3∶1) solvent system. Radioactive steroids were visualized by exposing TLC plates on imaging screens and reading them on a Fuji PhosphoImager FLA-7000 (Fujifilm, Dielsdorf, Germany). Specific steroids were identified according to known standards and densitometrically quantified using Multi Gauge software (Fujifilm). Steroid conversion was calculated as percentage of total radioactivity incorporated into specific products in relationship to total amount of radioactivity added.

### HSD3B2 Signaling Studies

NCI-R cells were cultivated in either growth or starvation medium on 12-well plates for 24 hrs. Treatments were added to fresh medium. Cells were treated with 0.5 mM 8Br-cAMP for 0 to 72 hours (short term –0 to 24 h; long term –24 to 72 h). The effect of 8Br-cAMP on HSD3B2 was studied by promoter luciferase assays, Western blots and steroid profiling as described above. Further signaling studies involved the following inhibitors: PD98059 MEK inhibitor (10 µM) [30], U0126 MEK inhibitor (10 µM) [31] and H89 PKA inhibitor (10 µM) [32]. We treated the NCI-R cells with PKA and MEK inhibitors in the presence and absence of 8Br-cAMP under normal (GM) or starved (SM) conditions for 48 hours before studying HSD3B2 expression and activity by Western blot and steroid profiling.

### Statistical Analysis

Statistical analysis was performed with the software Prism 4 (GraphPad Software, Inc. San Diego, CA) and Microsoft Windows Excel 2003. Statistical differences between values were calculated using the Student’s t test or the one-way ANOVA analysis. Quantitative data represent the mean of at least three independent experiments, error bars indicate the mean±SD. Significance was set at *p<0.05, and **p<0.01.

## Results

### Transcriptional Regulation of HSD3B2

**Figure 1:**
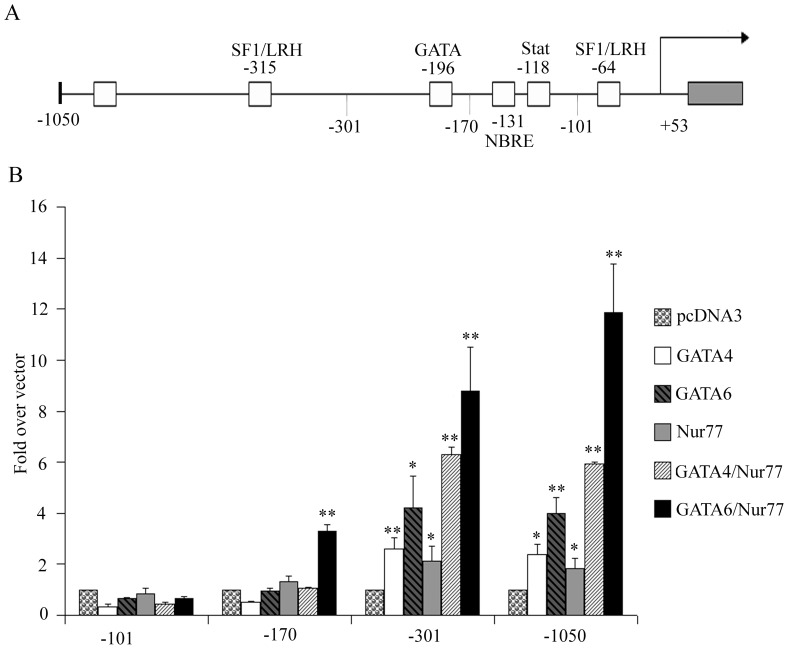
Essential transcription regulatory elements of the HSD3B2 promoter. (A) Diagram of the HSD3B2 promoter up to −1050 bp upstream with localization of identified transcription factor binding sites. (B) Activity of different HSD3B2 promoter reporter constructs when stimulated with different transcription factors. NCI-R cells were transiently transfected with HSD3B2 promoter luciferase reporter vectors and transcription factors GATA4/6 and Nur77 as indicated. Promoter activation was assessed by the dual luciferase assay (Promega) using pRL-TK as internal control. Essential activation of the HSD3B2 promoter by these factors seems to lie within the −301 HSD3B2 construct. Both GATA and Nur77 activate the promoter with strongest activation seen by GATA6/Nur77 in combination. Data are expressed as fold over vector control and given as mean±SD of three independent experiments performed in duplicate. * p<0.05, ** p<0.01.

The HSD3B2 gene is specifically expressed in the adrenals and the gonads. The promoter of HSD3B2 harbors binding sites for the transcription factors GATA [21], the NGFIB family [22], the liver receptor homolog (LRH1) [33], the steroidogenic factor 1 (SF1) [20] and the Stat family [25]. To study the transcriptional regulation of the human HSD3B2 gene, we prepared promoter reporter constructs of different length (Figure 1A) and assessed their activities in NCI-R cells upon transactivation with transcription factors GATA4/6 and Nur77. We found that for HSD3B2 promoter activation the essential regulatory elements appear to lie within the −301 to +53 bp of the gene (Figure 1B). Both GATA4/6 and Nur77 activated the −301 bp and the −1050 bp constructs similarly. Strongest activation was seen when GATA6 and Nur77 acted together on the HSD3B2 promoter constructs.

**Figure 2:**
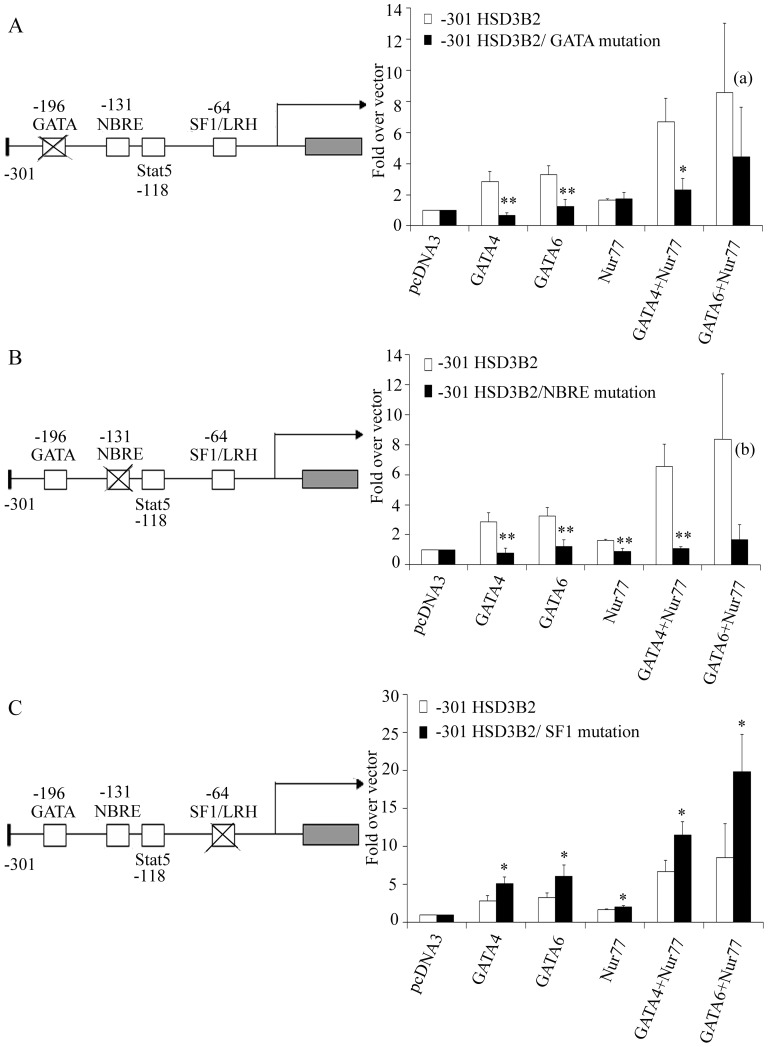
Role of transcription binding elements GATA, NBRE/Nur77 and SF1 for the activation of the HSD3B2 promoter in NCI-R cells. (A–C) Promoter activation by GATA and Nur77 was studied in NCI-R cells transiently transfected with −301 bp HSD3B2 promoter luciferase reporter constructs harboring specific binding site mutations. (A) Mutation of the GATA binding site in the −301 HSD3B2 construct showing loss of GATA induced promoter activation. (B) Mutation of the NBRE/Nur77 binding site revealing not only loss in Nur77 but also GATA4/6 induced promoter activation. (C) Mutation of the SF1/LRH binding site showing rather an increase in HSD3B2 promoter activation. Data are expressed as fold over vector control and given as mean±SD of three independent experiments. * p<0.05, ** p<0.01, (a) p = 0.26, (b) p = 0.060.

To further determine the role of GATA4/6, SF1 and Nur77 binding to the HSD3B2 promoter for its activity, we disrupted their specific binding sites in the −301 HSD3B2 promoter luciferase reporter constructs. Wild-type and mutant promoter reporters were then transfected into NCI-R cells and their activation by transcription factors GATA4/6 and Nur77 was assessed (Figure 2). Mutation of the GATA binding site in the HSD3B2 promoter led to a specific loss of the GATA4/6 induced promoter activation (Figure 2A). By contrast, mutation of the NBRE/Nur77 binding site revealed not only a loss in Nur77 transactivation but also caused a loss in GATA4/6 promoter transactivation suggesting an important collaboration between these two factors (Figure 2B). Conversely, mutation of the SF1 binding site rather increased transcriptional activation by GATA4/6, especially when combined with Nur77 (Figure 2C).

### GATA, SF1 and Nur77 are Essential for Tissue Specific HSD3B2 Expression

**Figure 3:**
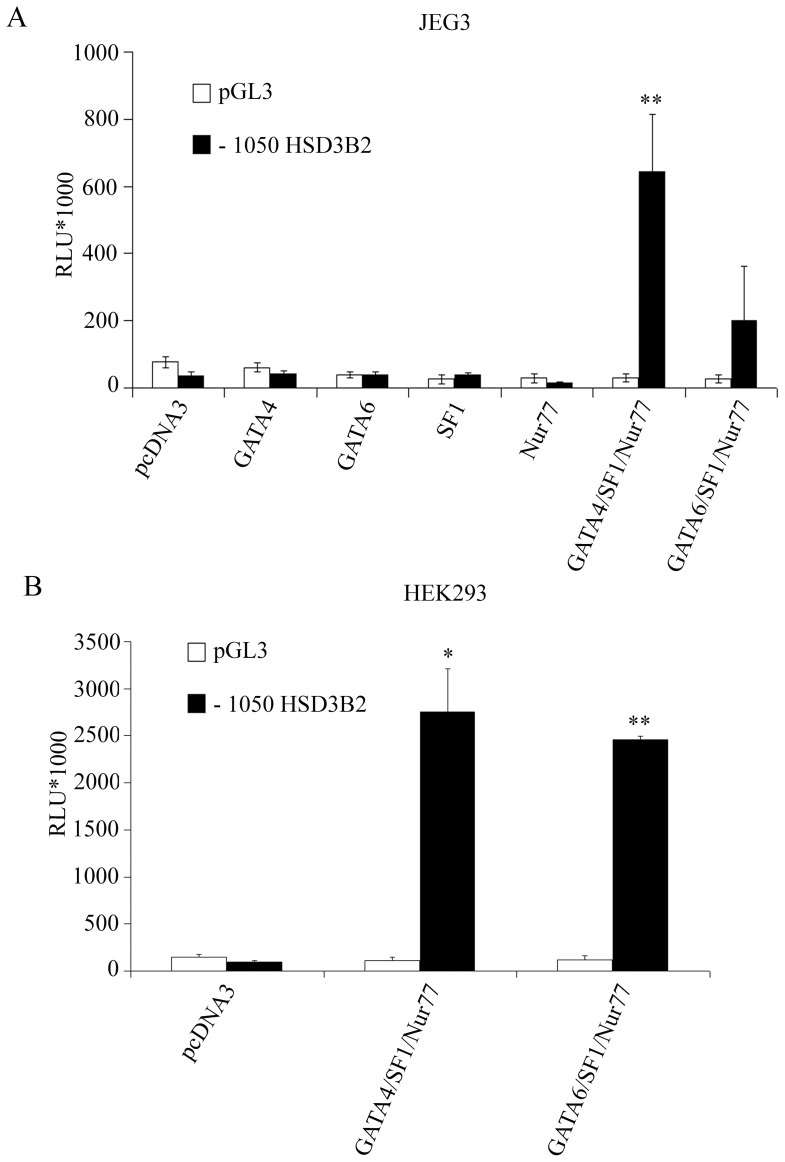
Identification of essential transcription factors for human HSD3B2 expression. Both steroidogenic human placental JEG3 and non-steroidgenic human embryonic kidney HEK293 cells (which do not express HSD3B2 normally) were transfected with the human −1050 HSD3B2 promoter construct and stimulated with different transcription factors. Promoter activation was assessed by the dual luciferase assay (Promega) using pRL-TK as internal control. (A) In JEG3 cells only transcription factors GATA, SF1 and Nur77 together are able to activate the HSD3B2 promoter. (B) Similar results are observed in non-steroidgenic HEK293 cells. Data are expressed in Relative Light Units (RLU). Error bars show the mean±SD of three independent experiments. * p<0.05, ** p<0.01.

Human HSD3B2 is expressed in the adrenal cortex and in the gonads but not in the placenta which expresses HSD3B1 for progesterone biosynthesis from pregnenolone [34], [35]. Although HSD3B1 and B2 are approximately 89% identical at the level of the DNA coding sequence and 93.5% identical at the protein level [2], the −1050 bp HSD3B2 promoter sequence differs significantly from the type 1 promoter. JEG3 cells do not express HSD3B2 and have a weak or no expression of the transcription factors GATA4/6, SF1 and Nur77 [27], [36]. We therefore hypothesized that those transcription factors might be essential for HSD3B2 expression. To address this question, we transfected JEG3 cells and non-steroidgenic HEK293 cells with the −1050 bp HSD3B2 promoter reporter construct and the candidate transcription factors (Figure 3). As predicted, the HSD3B2 promoter construct was inactive in both JEG3 and HEK293 cells (Figure 3A). Co-transfection with GATA4/6, SF1 or Nur77 alone was not able to activate the promoter construct. We observed a very significant activation of the HSD3B2 promoter only when all three transcription factors were combined. The control experiment in non-steroidogenic HEK293 cells revealed similar results (Figure 3B). Thus, we concluded that the transcription factors GATA4/6, SF1 and Nur77 work in concert to regulate HSD3B2 gene expression.

With these results, we wondered whether GATA4/6, SF1 and Nur77 would be also sufficient to activate the endogenous HSD3B2 gene in JEG3 or HEK293 cells. Therefore, we transfected these cells with GATA4/6, SF1 or Nur77 either alone or in combination and assessed endogenous HSD3B2 expression either by RT-PCR on total extracted RNA or by Western blot from protein extracts. While RT-PCR revealed inconsistent results, Western blots were clearly negative for endogenous HSD3B2 expression after transfection of JEG3 or HEK293 cells with GATA4/6, SF1 and Nur77 (data not shown).

Since some genes involved in steroidogenesis are regulated by epigenetic modifications [37], [38], [39], HSD3B2 was investigated for possible methylation and/or acetylation. JEG3 cells were treated with the histone methyltransferase inhibitor 5-aza-dC and/or the histone deacetylase inhibitor TSA and thereafter HSD3B2 as well as CYP17A1 expression (control) was assessed by quantitative RT-PCR as described [27], [40]. Neither 5-aza-dC nor TSA treatment was found to induce HSD3B2 expression in JEG3 cells (data not shown), while CYP17A1 expression was clearly inducible as previously reported [27]. These results indicate that the HSD3B2 gene is not regulated epigenetically.

### Effect of Serum Starvation on HSD3B2 in NCI-R Cells

**Figure 4:**
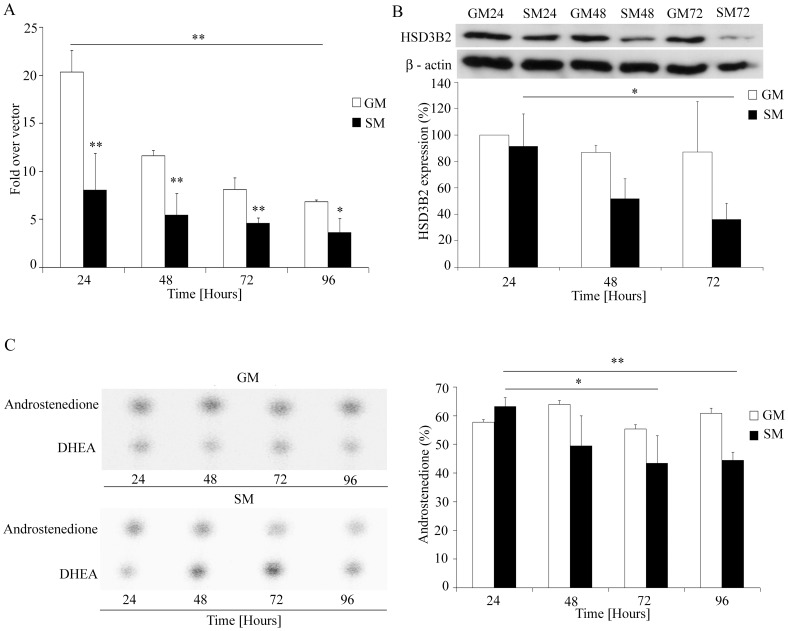
HSD3B2 expression and activity in NCI-R cells under normal and starvation growth conditions. (A) HSD3B2 promoter activity was assessed in NCI-R cells grown in normal growth medium (GM) and in medium without serum and ITS (starvation medium; SM) for 96 h. NCI-R cells were transiently transfected with empty vector and the −1050 HSD3B2 promoter luciferase construct. Promoter activation was assessed by the dual luciferase assay (Promega) using pRL-TK as internal control. Data are expressed as fold over vector control and error bars show the mean±SD of three independent experiments. (B) HSD3B2 expression under different growth conditions was assessed by Western blot analysis. Cells were grown for 24, 48, 72 hours in normal medium (GM) and in starvation medium (SM). β-actin served as loading control. Representative Western blots are shown. Quantitative results of three independent experiments are also depicted giving the mean±SD. (C) Activity of HSD3B2 for converting DHEA to androstenedione under either GM or SM growth conditions. NCI-R cells were grown for 96 h. Steroid production was labeled with [^3^H]DHEA for 60 min. Steroids were extracted and resolved by TLC. HSD3B2 activity was assessed by calculating the conversion of [^3^H]DHEA to [^3^H]androstenedione (%). A representative steroid profile (TLC) is shown (left) and the quantification of four independent experiments (mean±SD) is given (right). *p<0.05, **p<0.01.

The human adrenocortical NCI-R cell line is widely used for studies of steroidogenesis. NCI-R cells express HSD3B2 and produce all three types of adrenal steroid hormones, e.g. mineralocorticoids, glucocorticoids, and androgens [41]. NCI-R cells are usually cultured in growth medium containing 5% serum and insulin (www.atcc.org), but we and others observed that they can easily survive in serum free medium, and without insulin for at least 4 days [42], [43]. However, in our previous studies we have also observed that starvation conditions changed the steroid profile of NCI-R cells [44]. Therefore we aimed to investigate the differential regulation of HSD3B2 in NCI-R cells under normal growth and starvation conditions (Figure 4).

To test the impact of different growth conditions on HSD3B2 promoter activity, we transfected NCI-R cells with the −1050bp HSD3B2 promoter and grew the cells for up to 96 hours under normal growth or starvation conditions (Figure 4A). Compared to normal growth medium, starvation conditions caused a 60% loss of HSD3B2 promoter activity in NCI-R cells within the first 24 h. Overall, HSD3B2 promoter activity decreased under both normal growth and starvation conditions over time, but a more important decrease was observed for cells cultivated in normal growth medium over 96 h when they reached a promoter activity level similar to starvation conditions after 24 h (Figure 4A). At the protein level, we observed a significant decrease in HSD3B2 expression over 72 hrs under starvation conditions (Figure 4B). In addition, HSD3B2 expression was significantly lower under starvation (SM) than normal growth (GM) conditions (Figure 4B), a finding which fitted with the observed results for HSD3B2 promoter studies (Figure 4A). Finally, HSD3B2 activity was assessed in NCI-R cells grown for 96 h in both conditions. Conversion of labeled DHEA to androstenedione was compared (Figure 4C). Only under starvation conditions was a decrease in the conversion of DHEA to androstenedione and thus HSD3B2 activity observed. This decrease was significant after 72 h (−30%). In summary, all these experiments clearly showed that starvation led to an inhibition of HSD3B2.

Bearing in mind that timed HSD3B2 expression during fetal life is regulated by timed Nur77 expression [9], we also assessed Nur77 expression of NCI-R cells grown under starvation conditions. However, while starvation clearly decreased HSD3B2 expression after 24 hours, no such change was observed for Nur77 (data not shown).

### Regulation of HSD3B2 by cAMP

Adrenocorticotropic hormone (ACTH) is the most important regulator of adrenal glucocorticoid biosynthesis and a co-regulator of adrenal androgen production [45], [46], [47]. ACTH acts via its G-protein coupled receptor which predominantly activates the second messenger cAMP, and that in turn leads to the activation of the cAMP-dependent kinase (PKA) [45]. It is well recognized that stimulation of the ACTH/cAMP signaling pathway may have acute (short term) as well as chronic (long term) effects on steroidogenesis [47], [48], [49]. We investigated both the short and the long term effect of 8Br-cAMP stimulation on HSD3B2 in NCI-R cells.

**Figure 5:**
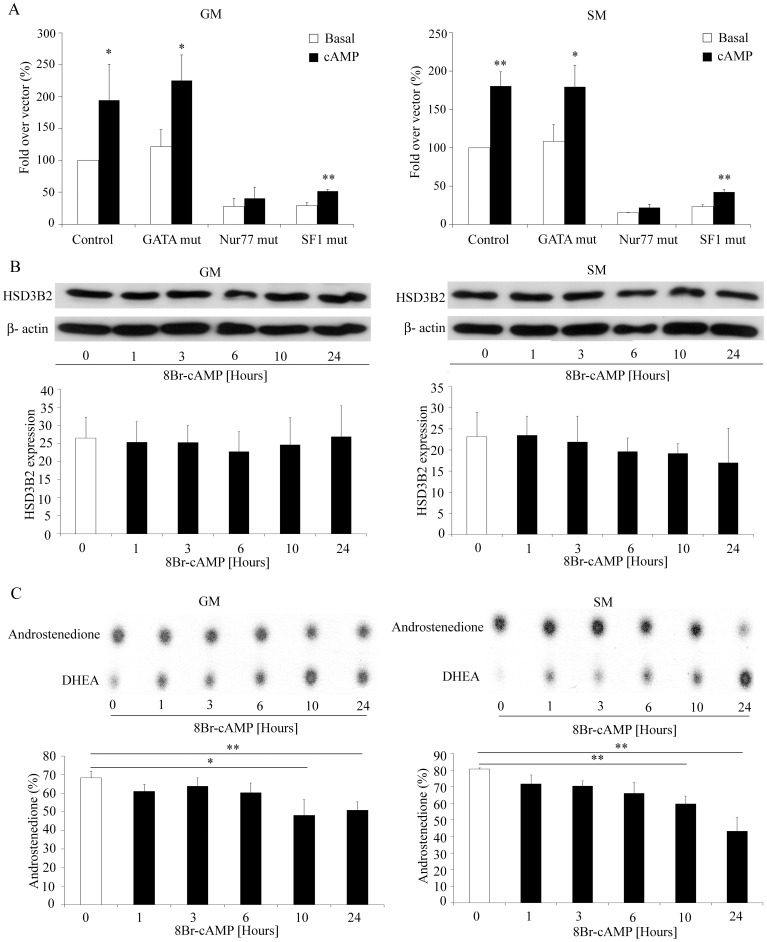
Regulation of HSD3B2 by 8Br-cAMP – short term. (A) NCI-R cells were transfected with the −301 HSD3B2 constructs with or without mutated GATA or NBRE/Nur77 transcription binding sites before stimulation with 0.5 mM 8Br-cAMP for 24 h under GM and SM growth conditions. Cyclic AMP stimulation of the HSD3B2 promoter seems to depend on an intact NBRE/Nur77 binding site. Data are expressed as fold over vector control (%) and error bars show the mean±SD of three independent experiments. (B) Effect of 8Br-cAMP stimulation on HSD3B2 expression was also assessed in NCI-R cells grown either under normal (GM) or starved (SM) conditions for 0–24 h. Expression was assessed by Western blot analysis using specific antibodies. β-actin served as loading control. Representative Western blots are shown. Quantitative results summarize three independent experiments (mean±SD). (C) HSD3B2 activity under 8Br-cAMP stimulation for up to 24 h. NCI-R cells were grown and treated with 0.5 mm 8Br-cAMP for 0–24 h under GM and SM growth conditions. The HSD3B2 activity for converting [^3^H]DHEA to [^3^H]androstenedione (%) was assessed. Steroids were extracted and resolved by TLC as depicted. Quantification of three independent experiments (mean±SD) is shown. *p<0.05, **p<0.01.

Short term effects: We studied the activity of the −301 bp HSD3B2 promoter construct in NCI-R cells cultured in normal growth or starvation medium with or without 8Br-cAMP stimulation for 24 h (Figure 5A). By mutating the GATA, NBRE/Nur77 or SF1 binding sites in the promoter constructs, we assessed which transcription factor might be involved in the cAMP targeted regulation. Eight Br-cAMP stimulation increased HSD3B2 promoter activity in either growth medium. Mutating the GATA binding site in the reporter construct did not abolish cAMP stimulation. By contrast, mutations of the NBRE/Nur77 and SF1 binding sites diminished basal promoter activity but only mutated NBRE/Nur77 diminished or abolished the cAMP effect (Figure 5A). Therefore, it appears that cAMP stimulation of the HSD3B2 promoter depends on an intact NBRE/Nur77 binding site.

Furthermore, we determined short term effects of 8Br-cAMP on protein expression and enzyme activity of HSD3B2 in NCI-R cells (Figure 5B–C). Cells were grown in different growth medium and stimulated with 8Br-cAMP for 0 to 24 h. Western blot analysis revealed no significant change in HSD3B2 protein expression after cAMP stimulation in both growth conditions (Figure 5B). HSD3B2 enzyme activity was assessed from the conversion of DHEA to androstenedione, and was found to decrease over time under both growth conditions (Figure 5C). Overall, after short term 8Br-cAMP stimulation, HSD3B2 DHEA to androstenedione conversion rate was lower in starvation medium than growth medium (SM 43% versus GM 51% after 24 h), and was lower after 8Br-cAMP stimulation and cell growth for 24 hours (Figure 5C) than after 24 h cell growth in either medium alone (Figure 4C) suggesting an additive short term inhibitory effect of starvation and 8Br-cAMP.

**Figure 6:**
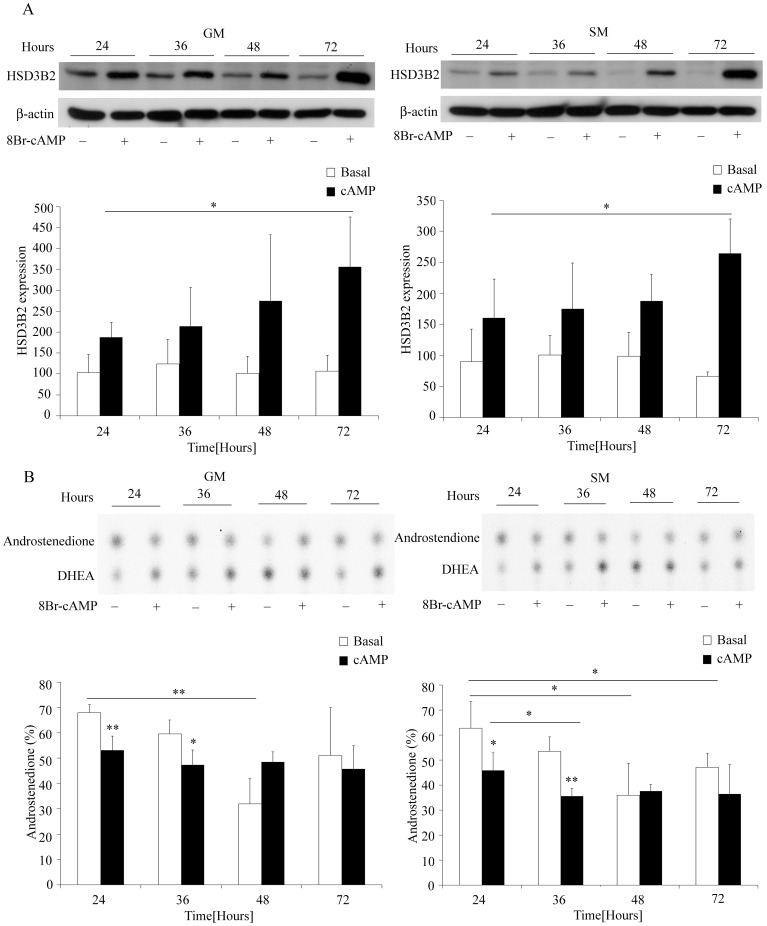
Regulation of HSD3B2 expression and activity by 8Br-cAMP – long term. (A) Effect of 8Br-cAMP stimulation on HSD3B2 expression was assessed in NCI-R cells grown either under normal (GM) or starved (SM) conditions for 24, 36, 48 and 72 hours. Expression was assessed by Western blot analysis using specific antibodies. β-actin served as loading control. Representative Western blots are shown. Quantitative results summarize three independent experiments (mean±SD). (B) HSD3B2 activity under 8Br-cAMP stimulation for up to 72 h. NCI-R cells were grown and treated with 0.5 mm 8Br-cAMP for 24, 36, 48 and 72 hours. HSD3B2 activity for converting [^3^H]DHEA to [^3^H]androstenedione (%) was assessed. Steroids were extracted and resolved by TLC as depicted. Quantification of three independent experiments (mean±SD) is shown. * p<0.05, **p<0.01.

Long term effects of cAMP: Similar to short term, the effect of long term (24–72 hours) 8Br-cAMP stimulation on protein expression and enzyme activity of HSD3B2 was studied in NCI-R cells under both growth and starvation conditions. We observed an increase in HSD3B2 expression after 24 h of 8Br-cAMP stimulation which was clearly significant after 72 h under both growth conditions (Figure 6A). HSD3B2 activity studies assessing conversion of DHEA to androstenedione revealed that without 8Br-cAMP (basal conditions) HSD3B2 activity significantly decreased over the first 48 h under both growth conditions and tended to increase thereafter. By contrast, 8Br-cAMP stimulation decreased HSD3B2 activity after 24 hours but led to no further loss of activity after 36 or 48 h for growth versus starvation conditions respectively (Figure 6B). Taken together long term (chronic) cAMP stimulation seems to increase HSD3B2 expression and support its activity.

### Role of PKA Signaling for HSD3B2 Regulation

**Figure 7:**
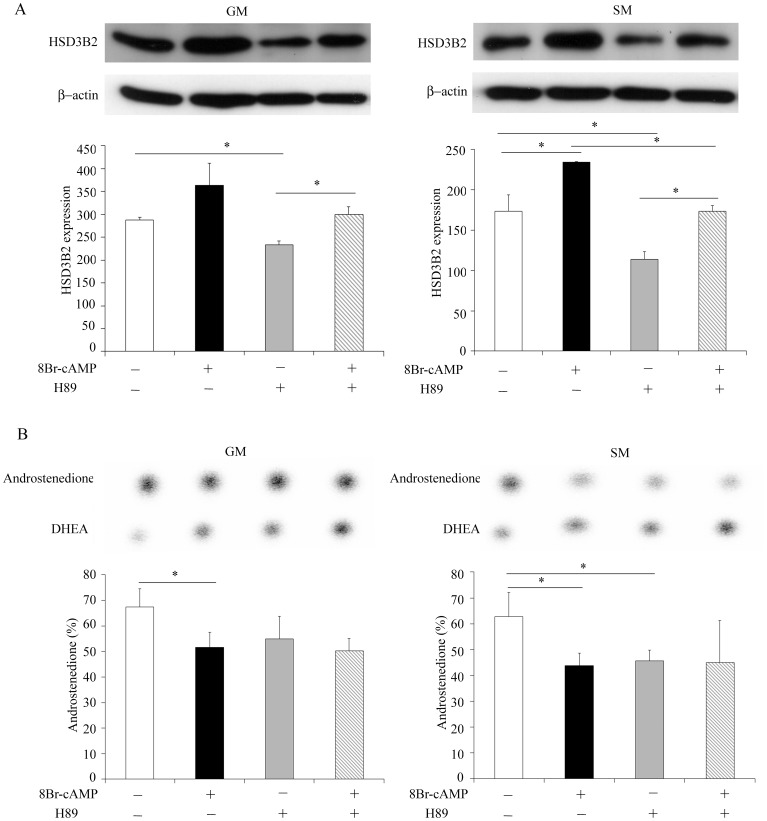
Role of PKA signaling for HSD3B2 expression and activity. (A) Effect of PKA inhibitor on HSD3B2 expression in presence and absence of 8Br-cAMP stimulation was assessed in NCI-R cells grown either under normal (GM) or starved (SM) conditions for 48 h. Expression was assessed by Western blot analysis using specific antibodies. β-actin served as loading control. Representative Western blots are shown. Quantitative results summarize three independent experiments (mean±SD). (B) HSD3B2 activity under PKA inhibition in presence and absence of 8Br-cAMP stimulation in NCI-R cells grown either in normal (GM) or starved (SM) conditions for 48 h. HSD3B2 activity for converting [3H]DHEA to [3H]androstenedione (%) was assessed. Steroids were extracted and resolved by TLC as depicted. Quantification of three independent experiments (mean±SD) is shown. * p<0.05.

It is well described that the ACTH/ACTH receptor complex activates the cAMP/PKA and the MEK/ERK1/2 signaling pathways in adrenal cells [45], [50] Therefore, after showing that HSD3B2 is clearly regulated by 8Br-cAMP (Figures 5, 6), we studied these signaling pathways in more detail. To determine the role of PKA on HSD3B2 regulation, we treated NCI-R cells with the PKA inhibitor H89 in the presence and absence of 8Br-cAMP under normal (GM) or starved (SM) conditions for 48 h. Western blot showed that under 8Br-cAMP stimulation HSD3B2 expression increased under both growth conditions (Figure 7A). By contrast, PKA inhibitor treatment significantly decreased the expression of HSD3B2 suggesting a role for PKA for HSD3B2 expression. However, the PKA inhibitor was not able to block cAMP stimulation on HSD3B2 indicating that the cAMP effect is not mediated through PKA signaling (Figure 7A). At the level of HSD3B2 activity, we found that 8Br-cAMP stimulation and H89 PKA inhibitor treatment caused a marked decrease in the conversion of DHEA to androstenedione under both cell growth conditions, but their effect was not additive (Figure 7B).

### Role of MAPK/ERK Signaling for HSD3B2 Regulation

**Figure 8:**
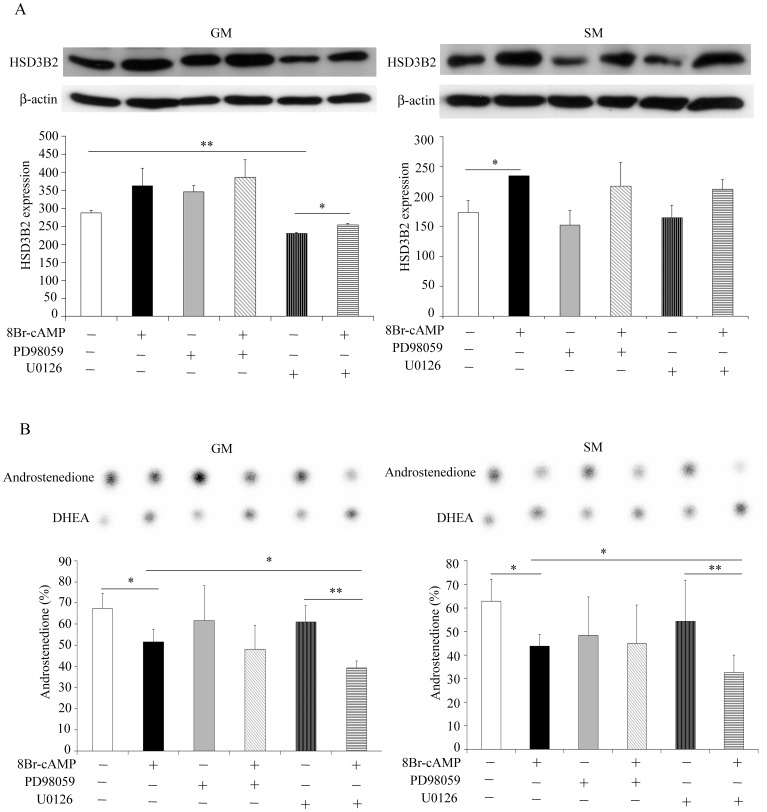
Role of MAPK/ERK1/2 signaling for HSD3B2 expression and activity. (A) Effect of MEK inhibitors on HSD3B2 expression in presence and absence of 8Br-cAMP stimulation assessed in NCI-R cells grown in normal (GM) or starvation (SM) medium for 48 h. Expression was assessed by Western blot analysis using specific antibodies. β-actin served as loading control. Representative Western blots are shown. Quantitative results summarize three independent experiments (mean±SD). (B) HSD3B2 activity under MEK inhibition in presence and absence 8Br-cAMP stimulation assessed in NCI-R cells grown either in normal (GM) or starved (SM) conditions for 48 h. The HSD3B2 activity for converting [3H]DHEA to [3H]androstenedione (%) was assessed. Steroids were extracted and resolved by TLC as depicted. Quantification of three independent experiments (mean±SD) is shown. * p<0.05, ** p<0.01.

Similar to PKA, we studied the effect of MAPK/ERK signaling on HSD3B2. NCI-R cells were treated with the MAPK/ERK inhibitors PD98059 and U0126 in presence and absence of 8Br-cAMP stimulation under normal (GM) or starved (SM) conditions for 48 h. PD98059 inhibits MEK1 [30] while U0126 inhibits both MEK1 and MEK2 kinases [31]. We observed that PD98059 inhibitor did not affect HSD3B2 expression under both growth conditions (Figure 8A). By contrast, U0126 inhibitor significantly decreased HSD3B2 expression especially under normal growth conditions. But cAMP stimulation was still effective under U0126 inhibitor treatment suggesting that cAMP does not employ MAPK/ERK signaling for its effect. Overall, effects were significant only under normal growth conditions but showed a similar pattern under starvation conditions (Figure 8A). HSD3B2 activity studies revealed no effect for PD98059 inhibitor treatment with and without 8Br-cAMP stimulation. But a significant decrease for DHEA to androstenedione conversion was found for U0126 inhibitor treatment under 8Br-cAMP stimulation (Figure 8B). This suggests an additive effect and precludes that the effect of cAMP is mediated through MAPK/ERK signaling.

## Discussion

In this study, we elucidated mechanisms regulating HSD3B2 for steroid hormone biosynthesis in human adrenal NCI-H295R cells. So far, it has been shown that the steroidogenic enzyme HSD3B2 which is essential for the production of all steroid hormones, is regulated at the transcriptional level by specific transcription factors [20], [21], [22], [25] and through hormonal stimulation such as ACTH and follicle stimulating hormone (FSH) [23], [51]. Previous studies reported that for transcriptional regulation of HSD3B2, GATA factors synergize with SF1 to activate the HSD3B2 promoter and stimulate steroidogenesis [21], [52]. Similarly, it has been shown that Nur77 and SF1/LRH1 co-activate the human HSD3B2 promoter and that all these transcription factors have their specific binding sites in the HSD3B2 promoter [22]. We now showed that all these factors work in concert to activate the promoter of the HSD3B2 gene. Accordingly, reporter constructs containing the regulatory elements of all these transcription factors are most active and combined overexpression of GATA together with Nur77 transcription factors enhance promoter activity most. Conversely, disrupting promoter binding sites of GATA and NBRE inhibits HSD3B2 promoter activity, and this is not only true for Nur77 stimulation but also for GATA stimulation when disrupting the NBRE site thereby suggesting a relevant collaboration between the transcription factors GATA and Nur77. By contrast, no such negative effect could be observed when destroying the SF1/LRH1 site at −64 bp in the promoter indicating that SF1/LRH1 regulate HSD3B2 indirectly through other regulatory complexes including GATA and Nur77. Overall our studies suggest that Nur77 predominantly is an essential transcriptional factor for HSD3B2 expression. In line with this, other investigators have also reported that the *cis*-element NBRE at −130 bp is both necessary and sufficient for Nur77 to activate the human HSD3B2 promoter in steroidogenic adrenal cells [22]. Therefore, we tested whether human HSD3B2 which is normally not expressed in placental JEG3 cells, might be transactivated through overexpression of Nur77 in JEG3 cells that have a weak or no expression of Nur77, SF1 and GATA4/6 [27], [36]. However, we found that Nur77 alone was not able to stimulate the HSD3B2 promoter, neither in JEG3 nor in non-steroidogenic HEK293 cells. Only overexpression of Nur77 with GATA and SF1 together seemed to stimulate the HSD3B2 promoter luciferase reporter. By contrast, overexpression of all these transcription factors failed in activating endogenous HSD3B2 expression. However, this negative result might be explained by too low transfection efficiency, so that expression levels of GATA/Nur77/SF1 in transfected cells were insufficient for activating endogenous HSD3B2. Expression levels of transcription factors are usually sufficient to induce effects when using reporter genes such as luciferase because this is a more sensitive endpoint. Alternatively, yet undiscovered gene expression control mechanisms may play a role.

Thus, we thought of additional epigenetic control of HSD3B2 expression as previously seen for the CYP17A1 gene which is similarly important for steroidogenesis in general and androgen production in particular (27). But our epigenetic studies revealed that neither acetylation nor methylation control the expression of the HSD3B2 gene in JEG3 cells.

Serum free starvation growth conditions have been shown to alter the steroid profile of NCI-R cells significantly by markedly increasing androgen production [44]. Therefore, we have been using the starved NCI-R cell model successfully for our studies of androgen regulation [40], [44], [53] or for studying mechanisms of action of androgen lowering drugs such as metformin [53]. Under starvation conditions a shift in the steroid profile of NCI-R cells towards DHEA and androstenedione synthesis was noted due to an increase of CYP17A1-17,20 lyase activity and a dramatic decrease of HSD3B2 activity [44]. As the physiologic event of adrenarche is also characterized by a decrease in HSD3B2 activity in the zona reticularis of the adrenal cortex [13], we remain interested in the regulatory events underlying HSD3B2 inhibition following starvation. Our new experiments revealed that this inhibition of HSD3B2 involves gene transcription, protein expression and function. Although it has been shown that specific expression of transcription factor Nur77 plays a crucial role for HSD3B2 expression during fetal adrenal development [9] and for adrenal zonation [24], starvation does not alter Nur77 expression in NCI-R cells. Most likely, Nur77 is therefore not the regulator of the starvation effect on HSD3B2.

ACTH is the key hormone regulating glucocorticoid and androgen biosynthesis of the adrenal cortex [1]; it also acts as a developmental and growth factor for the adrenals [54]. ACTH exerts its effect via a G-protein coupled receptor (melanocortin 2 receptor) which predominantly activates the second messenger cAMP, and that in turn leads to the activation of kinases which phosphorylate transcription factors regulating steroidogenesis [45], [47]. Cyclic AMP regulation of steroidogenesis appears to target many genes including steroidogenic acute regulatory protein StAR and CYP17A1 [47], [55], [56]. So far, little is known on the effect of cAMP on HSD3B2. We demonstrated that cAMP stimulates the HSD3B2 promoter. It appears that the cAMP dependent signaling targets the HSD3B2 promoter through the NBRE/Nur77 binding site. Previous studies on regulation of steroidogenesis have also suggested that Nur77 is most sensitive to hormonal regulation (e.g. luteinizing hormone (LH), ACTH and angiotensin II), although SF1 and LRH1 expression were also slightly affected [57], [58], [59], [60].

Generally, ACTH/cAMP stimulation may act on steroid hormone biosynthesis acutely (short term) or chronically (long term). Both the acute and chronic ACTH responses seem to be transmitted via cAMP-dependent signaling pathways and they occur in the order of minutes to hours [47]. Our study revealed that in response to short term cAMP stimulation HSD3B2 protein expression remained unaltered but HSD3B2 activity was inhibited indicating that there is an acute cAMP effect that bypasses gene transcription and protein expression. However, the exact (signaling) mechanism underlying this effect remains elusive. Possible mechanisms for direct HSD3B2 enzyme regulation which could be considered include the following: First, posttranslational modification of the HSD3B2 protein such as phosphorylation, glycosylation, ubiquitination and proteolysis changing enzyme amount or enzyme characteristics (substrate/co-factor specificity); second, cAMP might have a direct or indirect effect on co-factor and/or substrate availability for the enzyme. Finally, recently it has been shown that mitochondrial membrane translocases which associate with HSD3B2 are able to modulate enzyme activity through protein conformation change [61]. Most of these possible mechanisms need to be investigated for cAMP regulating HSD3B2 in future studies. Some experiments regarding protein degradation modulating basal and cAMP stimulated HSD3B2 expression using the proteasome inhibitor MG132 have been performed in our laboratory but revealed no change on protein expression (unpublished data).

By contrast, long term cAMP stimulation increases HSD3B2 expression after 24 hours resulting in a halt of HSD3B2 inhibition after 48 hours. Thus the long term effect of cAMP treatment is clearly at the level of HSD3B2 transcription and expression. In line with that, previous reports also show that cAMP stimulation modulates HSD3B2 expression through transcriptional regulation [62], [63].

ACTH/cAMP signaling in steroidogenesis is extremely complex and not fully understood [47]. Nevertheless a cross-talk between cAMP/PKA and other signaling pathways such as MEK/ERK1/2 or phosphatidyl-inositol 3-kinase/protein kinase B has been established by several studies [63], [64]. In mouse Leydig cells, LH and 8Br-cAMP have been shown to stimulate phosphorylation of ERK by PKA-dependent activation of Ras [65]. In our study we investigated cAMP regulated pathways PKA and MEK/ERK1/2 to determine their possible role in HSD3B2 regulation under different growth conditions. While inhibition of PKA is seen to inhibit HSD3B2 expression, it does not prevent cAMP from stimulating HSD3B2 indicating that cAMP does not (only) signal through this pathway. Similarly, inhibition of MEK1/2 results in a significant decrease of HSD3B2 expression without inhibiting the cAMP effect therefore suggesting that the MAPK pathway is also not crucial for cAMP signaling. Overall, both PKA and MAPK signaling pathways appear to modulate HSD3B2, however the main downstream signaling of cAMP to target HSD3B2 remains elusive. Moreover, observed effects of our signaling studies were similar under both normal and starvation growth conditions indicating that cAMP regulation as well as PKA and MEK/ERK pathways are not key modulators of the starvation cell program. Therefore, other regulators must be considered in future studies. Possible candidates include Src kinase (SRC), protein kinase C (PKC), and calcium-dependent calmodulin kinases (CaMK) which have been shown to mediate angiotensin II stimulation on HSD3B2 expression for aldosterone production through induction of rapid response genes (e.g. members of the NGFI-B family) as well as through phosphorylation of transcription factors and the cAMP-response element binding protein (CREB) [66].

In summary, through our studies we gained further insight into the regulation of HSD3B2 which plays a key role in steroidogenesis in health and disease. We confirm that HSD3B2 is specifically regulated at the transcriptional level with transcription factor Nur77 being essential but not sufficient for HSD3B2 expression. Importantly, short term cAMP stimulation seems to inhibit HSD3B2 activity directly while under long term cAMP stimulation HSD3B2 transcription and expression are increasing. Downstream signaling of cAMP does not depend on PKA or MEK/MAPK/ERK signaling although these pathways modulate HSD3B2 expression under basal conditions. Starvation inhibits HSD3B2 expression significantly by cellular mechanisms yet to be illustrated.
